# Genetic Diversity and Runs of Homozygosity in Three Masu Salmon (*Oncorhynchus masou*) Populations Based on Whole-Genome Resequencing Data

**DOI:** 10.3390/biom16081187

**Published:** 2026-08-14

**Authors:** Song Bai, Chenfan Geng, Wei Wang, Xiaoyu Yan, Tian Dong, Hailiang Song, Hongxia Hu

**Affiliations:** Fisheries Science Institute, Beijing Academy of Agriculture and Forestry Sciences & Beijing Key Laboratory of Fisheries Biotechnology, Beijing 100068, China; baisong@baafs.net.cn (S.B.); gengchenfan@baafs.net.cn (C.G.); wwei@baafs.net.cn (W.W.); yanxiaoyu@baafs.net.cn (X.Y.); dongtian@baafs.net.cn (T.D.)

**Keywords:** *Oncorhynchus masou*, whole-genome resequencing, genetic diversity, runs of homozygosity, genomic inbreeding

## Abstract

Masu salmon (*Oncorhynchus masou*) is an ecologically and economically important cold-water salmonid in East Asia that exhibits diverse life-history forms. To compare population-level genomic variation and patterns of homozygosity among fish from different sources, we analyzed whole-genome resequencing data from 465 individuals representing one field-collected Tumen River population (TM) and two landlocked cultured populations from Chicheng (CC) and Yanji (YJ). After quality control, 6,220,980 high-quality SNPs were retained. Population-specific filtering identified 5,589,828, 3,545,209, and 4,975,751 polymorphic SNPs in CC, TM, and YJ, respectively; although SNP numbers differed, approximately 91% of variants in each population were located in intronic or intergenic regions. Principal component analysis, ADMIXTURE, and distance-based neighbor-joining analysis clearly distinguished the three populations, with CC and YJ showing the closest genetic relationship. Pairwise $F_{\mathrm{ST}}$ was lowest between CC and YJ and highest between TM and YJ. CC exhibited the highest linkage disequilibrium, whereas TM showed the fastest LD decay and the lowest nucleotide diversity and heterozygosity. Runs of homozygosity (ROH) burden was highest in TM, intermediate in CC, and lowest in YJ. TM had the highest number of ROHs, cumulative ROH length, and $F_{\mathrm{ROH}}$, and ROHs longer than 5 Mb were detected only in this population. CC had an intermediate ROH burden dominated by short segments, whereas YJ had the lowest ROH-based genomic inbreeding. The high and heterogeneous ROH burden in TM indicates elevated genome-wide homozygosity among the sampled fish but does not, by itself, demonstrate recent inbreeding throughout the population. Candidate ROH islands and their annotated genes showed limited overlap among populations. Candidate genes in TM were primarily associated with ion regulation, neural processes, and energy metabolism, whereas those in CC and YJ shared broad functional categories involving development, muscle organization, nutrient transport, and neural regulation but differed in most specific genes. These regions and genes should be regarded as exploratory, hypothesis-generating candidates rather than evidence of selection or causality. Overall, this study reveals distinct population genomic characteristics and ROH patterns among masu salmon populations of different origins and provides a basis for future germplasm conservation and genetic management.

## 1. Introduction

Masu salmon (*Oncorhynchus masou*) is a cold-water salmonid of considerable economic, ecological, and conservation importance within the genus *Oncorhynchus*. As a Pacific salmon lineage endemic to Asia, it is distributed primarily across the Russian Far East, China, Japan, and the Korean Peninsula  [1,2]. The species exhibits substantial life-history diversity and has attracted considerable attention in fisheries management, aquaculture, conservation biology, and evolutionary ecology [3,4,5,6,7]. Owing to its desirable flesh quality, high market value, and adaptation to cold-water environments, masu salmon is an important resource for fisheries, stock enhancement, and aquaculture in East Asia [3,8,9].

Masu salmon exhibits marked life-history diversity, including anadromous and landlocked ecotypes. Anadromous individuals generally undergo early development in freshwater, experience smoltification and seaward migration, grow in the marine environment, and return to freshwater to spawn after reaching sexual maturity. In contrast, landlocked individuals complete their entire life cycle, including growth, maturation, and reproduction, in freshwater [1,10]. This life-history divergence is associated with pronounced differences in salinity tolerance, growth strategy, and physiological function. Before entering seawater, anadromous masu salmon undergo morphological and physiological changes associated with seawater adaptation, including body silvering, enhanced branchial ion-regulatory capacity, and endocrine adjustments that collectively improve survival and growth following seawater entry [4,11,12]. By contrast, long-term restriction to freshwater may reduce seawater tolerance in landlocked populations; nevertheless, appropriate salinity acclimation can confer some tolerance to seawater, suggesting potential for marine aquaculture [13]. Anadromous individuals can also exploit more abundant food resources in the marine environment and therefore tend to grow faster and reach a larger body size at maturity, whereas landlocked individuals exhibit growth and reproductive strategies adapted to freshwater habitats [14,15].

Advances in high-throughput sequencing and declining sequencing costs have made whole-genome resequencing an increasingly important tool for population genetic analysis, germplasm evaluation, and the genetic improvement of aquaculture species. Compared with traditional molecular markers, whole-genome resequencing provides high-density genetic variation across the genome, enabling higher-resolution analyses of genetic diversity, population structure, effective population size, and genomic signatures associated with artificial selection and domestication [16]. Runs of homozygosity (ROHs) are continuous homozygous genomic segments that generally arise when identical ancestral haplotypes are inherited from both parents; they therefore provide informative measures of individual autozygosity and genomic inbreeding [17,18]. The number, length, and genomic distribution of ROHs can reflect population history over different timescales. Long ROHs are commonly associated with recent common ancestry or mating in small populations, whereas shorter ROHs more often reflect distant common ancestry, genetic drift, or historical bottlenecks [19,20,21]. Genomic regions in which ROHs occur frequently across individuals, commonly termed ROH islands, may arise from artificial or natural selection or from locally reduced genetic diversity. They are therefore widely used to identify candidate genes potentially associated with domestication, adaptation, and economically important traits. ROH analyses have been applied to aquaculture species including Nile tilapia [22], rainbow trout [23,24], and coho salmon [25] to evaluate genomic inbreeding, changes in genetic diversity, and genomic patterns associated with selective breeding. However, although a chromosome-level reference genome and preliminary ROH estimates based on limited samples have recently been reported for masu salmon [7], comprehensive genome-wide comparisons of ROH patterns, genomic inbreeding, and ROH islands between field-collected and cultured landlocked populations remain lacking.

In this study, we used whole-genome resequencing data to compare population genetic structure, genetic diversity, and ROH patterns in one field-collected Tumen River population and two cultured landlocked populations of masu salmon. We first characterized genetic structure, differentiation, and within-population diversity and then assessed the number, length distribution, and genomic distribution of ROHs to estimate genomic inbreeding at both individual and population levels. We also identified population-specific candidate ROH islands and annotated their genes, focusing on regions with potential biological relevance to ion regulation, neural processes, energy metabolism, growth and development, and biological variation under culture conditions. Based on the documented source relationship, we hypothesized that CC and YJ would be more closely related to each other than either would be to TM. We further expected the three sampled populations to differ in genetic diversity and ROH profiles because of their distinct origins and population histories. These analyses provide new insights into genetic differentiation, genomic inbreeding, and candidate ROH islands in field-collected and cultured landlocked masu salmon populations and offer genomic information relevant to germplasm conservation and genetic management.

## 2. Materials and Methods

### 2.1. Population

A total of 465 masu salmon (*Oncorhynchus masou*) from three populations were analyzed. The Yanji (YJ) population comprised 96 cultured individuals sampled in October 2023 from the Yanbian Masu Salmon Improved Variety Farm in Xiawazi Village, Mijiang Township, Hunchun City, Yanbian Korean Autonomous Prefecture, Jilin Province, China. This population was established from wild masu salmon collected from the Tumen River between 2012 and 2015 and was subsequently maintained through captive propagation. The Chicheng (CC) population comprised 273 cultured individuals sampled in October 2023 from Zhangjiakou Juxunyuan Fishery Co., Ltd., in Yunzhou Village, Yunzhou Township, Chicheng County, Zhangjiakou City, Hebei Province, China. CC was established from approximately 10,000 juveniles introduced from the Yanbian Masu Salmon Improved Variety Farm in June 2023, when their mean body weight was 2 g. The Tumen (TM) population comprised 96 field-collected individuals sampled from a natural spawning ground for masu salmon in the Tumen River, Jilin Province, China, in October 2023.

The original sampling records for all 465 individuals, including population identity, body weight, available fork length and recorded sex information, are provided in Supplementary Table S1. Mean body weight was 34.88 ± 7.43 g in CC, 2341.25 ± 537.60 g in TM, and 559.48 ± 240.20 g in YJ.

Fin clips were collected from each individual and preserved in ethanol until DNA extraction. Genomic DNA was extracted using a QIAGEN DNeasy Blood and Tissue Kit (QIAGEN, Hilden, Germany) according to the manufacturer’s instructions. DNA quality was assessed by agarose gel electrophoresis, and the extracted DNA was used for whole-genome resequencing. The use of the archived samples and the associated research procedures was subsequently reviewed and approved by the Ethics Committee of Beijing Academy of Agriculture and Forestry Sciences (protocol code BAAFS-KJLL-20250909; 9 September 2025).

### 2.2. Genotyping and Quality Control

All 465 individuals were sequenced in a single batch on an Illumina platform using 150-bp paired-end reads (PE150). Raw reads were filtered and trimmed with fastp v0.23.4 [26]. An entire read pair was discarded if either mate contained undetermined bases (N) at more than 10% of positions, if bases with a Phred quality score of Q ≤ 5 accounted for more than 50% of either mate, or if adapter contamination was detected. Read pairs retained after filtering were designated clean reads and used for alignment and variant discovery. Clean reads were aligned to the masu salmon reference genome (NCBI assembly UVic_Omas_1.1; accession GCF_036934945.1) [7] using the BWA-MEM algorithm in BWA v0.7.17 [27]. Alignment files were converted to BAM format and sorted by genomic coordinates using SAMtools v1.21 [28]. PCR and optical duplicates were identified and marked using MarkDuplicates in GATK v4.3 [29]. No additional read-level mapping-quality threshold was applied during BAM conversion; mapping quality was considered subsequently during site-level variant filtering.

Variant calling was performed using GATK v4.3 [29]. For each individual, HaplotypeCaller was run in GVCF mode (-ERC GVCF) on the coordinate-sorted, duplicate-marked BAM file. Individual GVCF files from all 465 samples were combined by chromosome with CombineGVCFs and jointly genotyped with GenotypeGVCFs. For each chromosome, SNPs were extracted from the jointly genotyped VCF using SelectVariants with –select-type SNP and –exclude-non-variants. Raw SNPs were hard-filtered with VariantFiltration using the following criteria: QD < 2.0, MQ < 40.0, FS > 60.0, SOR > 3.0, MQRankSum < −12.5, and ReadPosRankSum < −8.0. Filtering was performed separately for each chromosome, with criteria combined using the logical OR operator. Variants passing all site-level criteria were retained with –exclude-filtered, and the filtered chromosome-level files were merged into a genome-wide SNP dataset. No additional filters were applied at this stage. SNP-level and individual-level quality control was then performed in PLINK v1.90 [30] using a minor allele frequency (MAF) threshold of >0.05, variant missingness of <0.05, a Hardy–Weinberg equilibrium test threshold of $p>1\times 10^{-6}$, and individual missingness of <0.10. The corresponding options were –snps-only, –mind 0.10, –geno 0.05, –maf 0.05, and –hwe $1\times 10^{-6}$ . MAF and Hardy–Weinberg equilibrium were calculated jointly across all 465 individuals. For population-specific SNP-distribution analyses, sites that were monomorphic within a population were excluded to generate population-specific polymorphic SNP datasets. The chromosomal distribution and genomic context of these SNPs were characterized using BEDTools [31] and the GFF3 annotation of the UVic_Omas_1.1 reference genome. SNPs were classified as occurring in coding sequences (CDSs), other exonic regions, splice sites, introns, upstream/downstream regions, or intergenic regions. SNP number and density were also calculated for chromosomes 1–33 using chromosome lengths from the reference assembly.

### 2.3. Population Structure Analyses

Population structure was investigated using principal component analysis (PCA), model-based ancestry inference, and a distance-based neighbor-joining (NJ) tree. All three analyses used the same PLINK-filtered dataset of 6,220,980 SNPs from 465 individuals without linkage disequilibrium (LD) pruning. PCA was performed using Genome-wide Complex Trait Analysis (GCTA) v1.94.1 [32]. A genomic relationship matrix was first constructed from autosomal SNPs with –make-grm, and 20 principal components were then calculated with –pca 20. The first two principal components were used to visualize genetic differentiation among individuals and populations. Individual ancestry proportions were estimated using ADMIXTURE v1.3.0 [33] for K = 2–5, with cross-validation performed using –cv. Genetic relationships among individuals were further examined using an NJ tree. Pairwise genetic distances based on identity-by-state (IBS) were calculated in PLINK v1.90 [30] with –distance square 1-ibs. The resulting matrix was converted to an R distance object, and the NJ tree was constructed using the nj function in ape v5.8-1 [34]. The tree was displayed in a circular fan layout using ggtree, with individuals colored by sampling population. Because no bootstrap or other branch-support analysis was performed, the NJ tree was interpreted as a distance-based visualization of genetic relationships rather than a supported phylogenetic reconstruction. The PLINK-filtered dataset was converted to VCF format using –recode vcf. LD decay was evaluated separately in CC, TM, and YJ using PopLDdecay v3.43 [35]. The same genome-wide SNP dataset was used for each population, with individuals selected using population-specific sample lists supplied through -SubPop. Because no other parameters were specified, the default settings were used: a maximum SNP-pair distance of 300 kb (-MaxDist 300), a minimum MAF of 0.005 (-MAF 0.005), a maximum heterozygous-site proportion of 0.88 (-Het 0.88), and a maximum missing-site proportion of 0.25 (-Miss 0.25). Output type 1 was used for the plotted mean $r^{2}$ curve, and output type 5 was also generated to retain summed $r^{2}$ values and SNP-pair counts at each distance. For numerical summaries, distances were grouped into successive 1-kb bins, and pair-weighted mean $r^{2}$ was calculated by dividing the summed $r^{2}$ values by the total number of SNP pairs. Short-range LD was represented by the 1–1000-bp bin. A relative half-decay distance was estimated by linear interpolation between adjacent 1-kb-bin midpoints at which pair-weighted $r^{2}$ crossed one-half of this short-range value. These summaries were used to characterize population-specific LD decay.

### 2.4. Genetic Diversity Analyses

Within-population genetic diversity was assessed using nucleotide diversity (π) and heterozygosity. All diversity and differentiation analyses used the quality-controlled SNP dataset retained after PLINK filtering. Nucleotide diversity was calculated separately for CC, TM, and YJ using VCFtools v0.1.16 [36]. Population-specific sample lists were supplied for each analysis, and π was calculated in 50-kb windows with a 25-kb step using –window-pi 50000 and –window-pi-step 25000. Genetic differentiation was evaluated for CC–TM, CC–YJ, and TM–YJ using the Weir–Cockerham $F_{\mathrm{ST}}$ estimator in VCFtools. For each comparison, sample lists for the two populations were supplied using –weir-fst-pop, and windowed $F_{\mathrm{ST}}$ was calculated with –fst-window-size 50000 and –fst-window-step 25000. Thus, both π and $F_{\mathrm{ST}}$ were estimated in 50-kb windows with a 25-kb step [37,38]. Windows containing at least one valid variant and a finite π or weighted $F_{\mathrm{ST}}$ estimate were retained as effective windows. Genome-wide mean π and the mean of the window-level weighted Weir–Cockerham $F_{\mathrm{ST}}$ estimates were calculated across effective windows. Uncertainty was evaluated using a leave-one-chromosome-out block jackknife. Each of the 33 chromosomes was excluded in turn, the statistic was recalculated from windows on the remaining chromosomes, and jackknife standard errors and 95% confidence intervals (CIs) were calculated from the 33 estimates. Because the input was a filtered SNP-only VCF, invariant callable positions were not explicitly represented; π therefore describes diversity estimated from the retained SNP dataset rather than a callability-controlled genome-wide estimate. Observed heterozygosity ($H_{o}$) and expected heterozygosity ($H_{e}$) were calculated separately for each population using –het in PLINK v1.90 [30]. For each individual, $H_{o}=\left[N_{\mathrm{NM}}-O\left(\mathrm{HOM}\right)\right]/N_{\mathrm{NM}}$, where O(HOM) is the observed number of homozygous genotypes and $N_{\mathrm{NM}}$ is the number of non-missing genotypes. Expected heterozygosity was calculated as $H_{e}=\left[N_{\mathrm{NM}}-E\left(\mathrm{HOM}\right)\right]/N_{\mathrm{NM}}$, where E(HOM) is the expected number of homozygous genotypes based on population-specific allele frequencies. Population-level $H_{o}$ and $H_{e}$ were obtained by averaging individual estimates within each population. These estimates describe heterozygosity across the retained polymorphic SNPs rather than unrestricted genome-wide heterozygosity.

### 2.5. Runs of Homozygosity

Runs of homozygosity (ROHs) were identified separately in CC, TM, and YJ using –homozyg in PLINK v1.90 [30]. The same quality-controlled SNP dataset and identical detection parameters were used for all three populations: –homozyg-density 50, –homozyg-gap 100, –homozyg-kb 350, –homozyg-snp 50, –homozyg-window-snp 50, –homozyg-window-het 1, –homozyg-window-missing 2, and –homozyg-window-threshold 0.05. An ROH was therefore defined as a continuous homozygous genomic segment at least 350 kb long and containing at least 50 SNPs, with an average marker spacing no greater than 50 kb and no gap greater than 100 kb between consecutive SNPs. Scanning was performed in 50-SNP windows, with no more than one heterozygous and two missing genotypes permitted per window. These allowances applied to each scanning window rather than to the entire ROH; consequently, heterozygous genotypes were not completely excluded from detected ROHs. A SNP was eligible for inclusion in an ROH if at least 5% of the overlapping windows containing that SNP were classified as homozygous.

To characterize the distribution of genomic homozygosity, ROHs were assigned to seven length categories: 0.35–0.5 Mb, 0.5–1 Mb, 1–2 Mb, 2–3 Mb, 3–4 Mb, 4–5 Mb, and >5 Mb. Segment lengths generated by PLINK were converted from kilobases to megabases and assigned to the corresponding category. The number and cumulative length of ROHs in each category were calculated for each population. To compare length distributions among populations, the cumulative length within each category was divided by the cumulative length of all ROHs detected in that population and expressed as a percentage. The stacked bar plot therefore represents the contribution of each length category to the population’s cumulative ROH length, rather than the proportion of individuals or segments. The number of ROHs and cumulative ROH length were also summarized for each individual to assess within- and among-population variation. Mean ROH length was calculated as cumulative ROH length divided by the number of detected segments. Individual-level ROH traits were summarized for each population using the mean ± standard deviation (SD), median and first and third quartiles [median (Q1–Q3)], interquartile range (IQR), and minimum–maximum range. Individuals without detected ROHs were retained and assigned values of zero for ROH number, cumulative ROH length, and $F_{\mathrm{ROH}}$. Because mean ROH length is undefined when no ROH is detected, this metric was summarized only for ROH-positive individuals. Full individual-level distributions were visualized using violin plots overlaid with boxplots and individual data points.

Because individual ROH number, cumulative ROH length, and $F_{\mathrm{ROH}}$ were strongly skewed and population sample sizes were unequal, differences among the three sampled populations were evaluated using Kruskal–Wallis tests. Significant omnibus tests were followed by two-sided pairwise Mann–Whitney *U* tests with Holm correction. Overall effect size was summarized using epsilon-squared ($\epsilon^{2}$), and pairwise effects were summarized using rank-biserial correlations. An outlier-sensitivity analysis was also performed for TM. Upper outliers were defined as $F_{\mathrm{ROH}}>Q3+1.5\times \mathrm{IQR}$; 17 TM individuals met this criterion, including the individual with the maximum $F_{\mathrm{ROH}}$. The omnibus and pairwise tests were repeated after excluding these individuals. These sample-level tests were not adjusted for unknown kinship. Statistical analyses were performed in Python 3.13.11 using SciPy 1.17.0.

### 2.6. Estimation of Inbreeding Coefficients

ROH-based genomic inbreeding coefficients ($F_{\mathrm{ROH}}$) were estimated following McQuillan et al. [17]. For each individual, $F_{\mathrm{ROH}}$ was calculated as:

(1) $$F_{i,\mathrm{ROH}}=\frac{\sum L_{\mathrm{ROH},i}}{L_{\mathrm{Genome}}},$$

where $\sum L_{\mathrm{ROH},i}$ is the cumulative length of all ROHs detected in individual *i*, and $L_{\mathrm{Genome}}$ is the chromosome-level reference-genome length used as a common operational denominator. In this study, $L_{\mathrm{Genome}}$ was set to 2.7 Gb based on the assembled masu salmon genome [7]. This fixed denominator was not recalculated from an autosomal callability mask; it therefore supports consistent relative comparisons among individuals but may affect the absolute scale of $F_{\mathrm{ROH}}$. Individual $F_{\mathrm{ROH}}$ values were calculated from the PLINK output and summarized by population to compare genomic inbreeding levels.

### 2.7. Identification and Functional Annotation of Candidate ROH Islands

ROH islands, defined as genomic regions encompassed by ROHs in many individuals from the same population, were identified from SNP-level ROH incidence [39]. For each population and SNP, we counted the number of individuals in which the SNP was encompassed by an ROH. This count was divided by the number of individuals in that population with at least one detected ROH and multiplied by 100. Accordingly, ROH incidence represents a conditional percentage among ROH-positive individuals rather than prevalence among all sampled individuals.

For each population independently, SNPs in the top 0.1% of the genome-wide ROH-incidence distribution were retained as high-incidence candidate SNPs, including all SNPs tied at the cutoff [24,40]. Adjacent candidate SNPs were then clustered by physical proximity, with neighboring signals separated by no more than 100 kb merged into a single candidate ROH island.

To evaluate the sensitivity of the population-specific top-0.1% criterion, SNP-level ROH prevalence was also recalculated using all sampled individuals in each population as the denominator (CC, 273; TM, 96; YJ, 96). Common minimum prevalence thresholds of 5%, 10%, and 20% were then applied to every population, corresponding to minimum counts of 14, 28, and 55 individuals in CC and 5, 10, and 20 individuals in TM and YJ. Candidate SNPs passing each fixed threshold were merged using the same ≤100-kb distance rule, and regions containing fewer than five candidate SNPs were excluded. This analysis was used only to assess threshold sensitivity and was not used for functional annotation.

For each island, the chromosome and start and end coordinates were defined by the minimum and maximum physical positions of its constituent SNPs. number of candidate SNPs, island length, and mean and maximum ROH incidence were calculated to describe the extent and strength of each homozygosity signal. Only islands containing at least five SNPs were retained for functional annotation. These regions are hereafter referred to as candidate ROH islands.

Genomic annotations overlapping candidate ROH islands were identified using intersect -wa in BEDTools v2.31.1 [31]. The GFF3 file for the UVic_Omas_1.1 reference assembly (GCF_036934945.1) served as the annotation source. Unique gene identifiers were extracted from overlapping GFF3 records and used to retrieve the corresponding XP protein sequences from the protein FASTA file associated with the same annotation.

Functional annotation was performed using eggNOG-mapper v2.1.12 [41], with protein sequences queried against the locally installed eggNOG database emapperdb-5.0.2 [42] using DIAMOND. The DIAMOND search used the sensitive iterative mode, an E-value threshold of $1\times 10^{-3}$, and the top three target matches. Orthology assignments and functional descriptions were recorded at the XP protein-accession level, while the correspondence between each XP accession and its source gene identifier was retained. Although all XP accessions associated with each gene were annotated, only the eggNOG-mapper result for the first XP accession was retained for manual interpretation and is reported in Supplementary Table S7, together with the genomic coordinates of each gene and the identifier and coordinates of its corresponding candidate ROH island; multiple accessions from the same gene were therefore not treated as independent candidates. Supplementary Table S6 reports all candidate ROH islands and their numbers of overlapping reference genes, whereas Supplementary Table S7 provides the complete gene-to-island coordinate mapping and functional annotation output. The occurrence of a gene within a candidate ROH island was considered exploratory evidence of possible biological relevance.

## 3. Results

### 3.1. Sequencing Quality, SNP Filtering, and Genomic Distribution

After read filtering, each individual retained an average of 75.93 million clean read pairs and 21.86 Gb of clean sequence data, with ranges of 47.18–197.13 million read pairs and 13.46–57.46 Gb, respectively. Mean read length, Q20, Q30, GC content, and the proportion of undetermined bases were 143.90 bp, 98.51%, 96.12%, 41.97%, and 0.0095%, respectively. Detailed population- and sample-level clean-read quality-control metrics are provided in Supplementary Tables S2 and S3.

Before PLINK filtering, the genome-wide dataset contained 125,475,462 variants across 465 individuals, with an overall genotype call rate of 99.6238%. No individual was removed by the missingness filter. SNP-level missingness, Hardy–Weinberg equilibrium, and MAF filters removed 2,130,071, 49,212,690, and 67,911,721 variants, respectively. After all filtering steps, 6,220,980 high-quality SNPs from all 465 individuals were retained for downstream analyses.

Population-specific filtering retained 5,589,828, 3,545,209, and 4,975,751 polymorphic SNPs in CC, TM, and YJ, respectively. SNPs were distributed across all 33 chromosomes, with chromosome-level counts generally increasing with chromosome length. Overall SNP densities were approximately 2568, 1629, and 2286 SNPs/Mb in CC, TM, and YJ, respectively.

The proportional distribution of SNPs among genomic annotation categories was highly similar across populations. Intronic SNPs accounted for 46.16%, 45.83%, and 46.21% of polymorphic SNPs in CC, TM, and YJ, respectively, whereas intergenic SNPs accounted for 45.20%, 44.84%, and 45.23%. Together, intronic and intergenic variants represented approximately 91% of SNPs in each population. Other exonic variants accounted for 3.54–3.70%, upstream/downstream variants for 3.32–3.34%, and CDS variants for 1.68–2.26%. Variants within 2 bp of annotated exon boundaries represented less than 0.03% of SNPs in all three populations. Thus, despite marked differences in the number of polymorphic SNPs, their proportional distribution among genomic annotation categories was consistent across populations. Chromosome-specific SNP counts and densities and genomic-category summaries are provided in Supplementary Tables S4 and S5, respectively.

### 3.2. Population Structure

**Figure 1:**
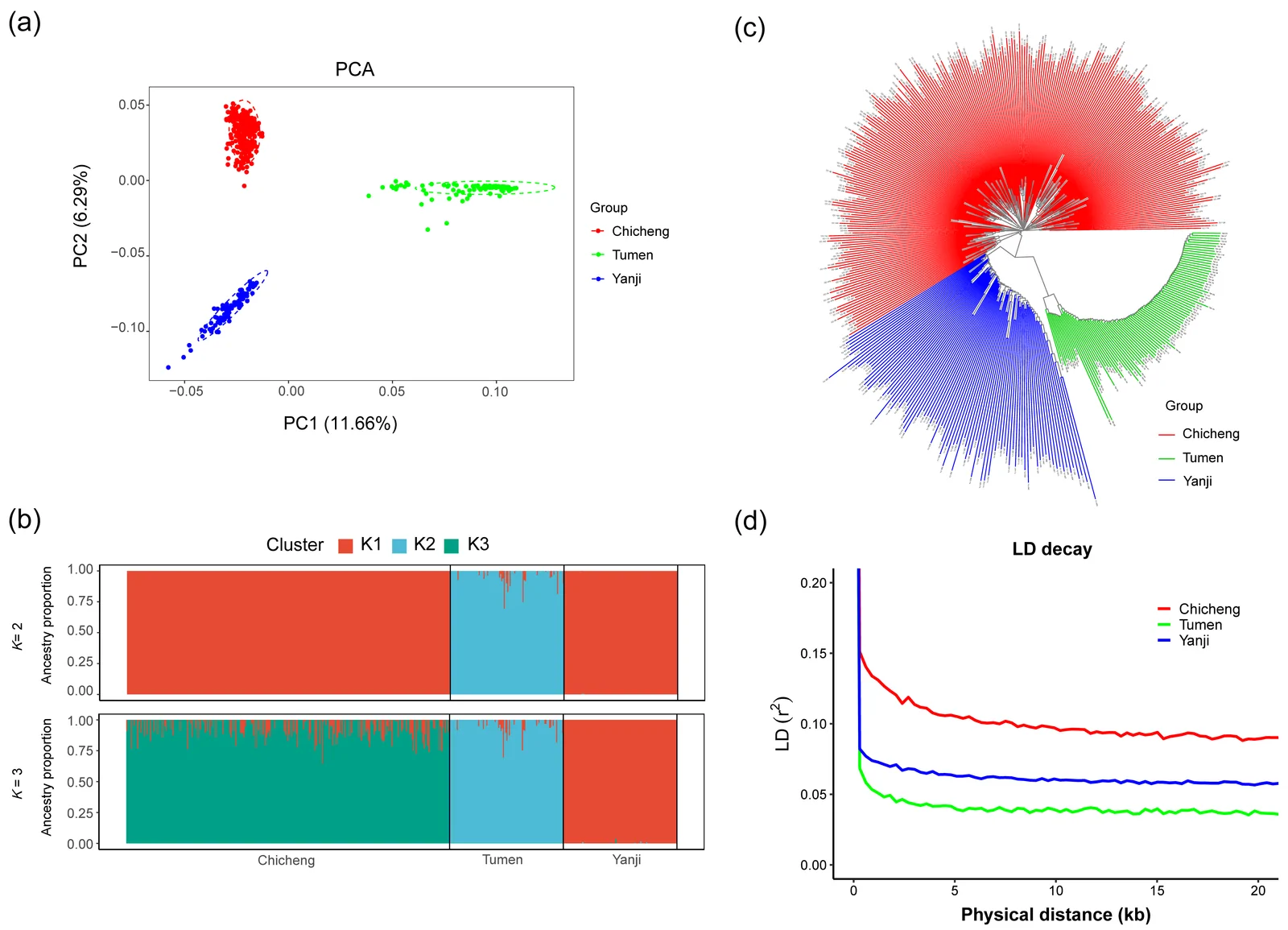
Genetic structure and linkage disequilibrium patterns in three masu salmon populations. (**a**) Principal component analysis (PCA) of individuals from Chicheng (CC), Tumen (TM), and Yanji (YJ) based on genome-wide SNPs. (**b**) Model-based ancestry proportions inferred with ADMIXTURE at K = 2 and K = 3. (**c**) Distance-based neighbor-joining (NJ) tree showing genetic relationships among individuals. (**d**) Linkage disequilibrium (LD) decay, measured as mean pairwise $r^{2}$, plotted against physical distance (kb) for each population. Red, green, and blue represent CC, TM, and YJ, respectively, in panels (**a**), (**c**), and (**d**).

Principal component analysis (PCA) revealed clear genetic separation among the three masu salmon populations (Figure 1a). The field-collected Tumen River population (TM) was separated from the two landlocked cultured populations, Chicheng (CC) and Yanji (YJ), along the first principal component (PC1), which explained 11.66% of the variance. CC and YJ were further separated along PC2, which explained 6.29% of the variance.

ADMIXTURE analysis supported the PCA results (Figure 1b). Cross-validation errors were 0.45542, 0.26144, 0.25782, and 0.25521 for K = 2, 3, 4, and 5, respectively. Although the lowest error occurred at K = 5, K = 2 and K = 3 captured the principal levels of population structure. The complete ADMIXTURE results for K = 2–5 are provided in Figure S1. At K = 2, individuals were assigned primarily to two ancestry components corresponding to TM and the two landlocked cultured populations (CC and YJ). At K = 3, the three sampled populations were represented by distinct ancestry components; most individuals had a high proportion of population-specific ancestry, with only limited admixture signals.

The distance-based NJ tree showed a clustering pattern consistent with the PCA and ADMIXTURE results (Figure 1c). Individuals clustered by sampling population: TM formed a group distinct from the two cultured populations, whereas CC and YJ formed separate clusters within the landlocked group.

All three populations exhibited rapid LD decay (Figure 1d). The relative half-decay distances were 21.4, 1.27, and 6.53 kb in CC, TM, and YJ, respectively. Accordingly, CC showed the slowest LD decay, YJ was intermediate, and TM showed the fastest decay. Complete numerical summaries are provided in Supplementary Table S9.

Overall, PCA, ADMIXTURE, and NJ analyses consistently distinguished the three populations, while the LD decay profiles revealed additional differences in genome-wide linkage structure.

### 3.3. Genetic Differentiation and Diversity

**Figure 2:**
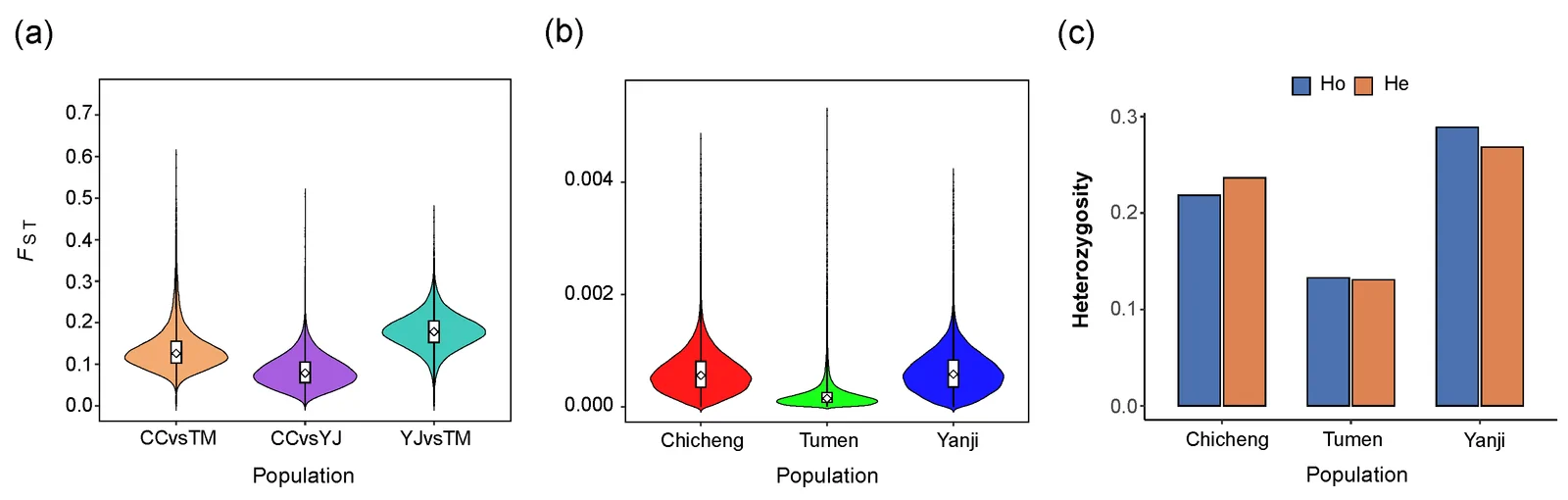
Genetic differentiation and diversity in three masu salmon populations. (**a**) Distribution of window-based $F_{\mathrm{ST}}$ values for CC–TM, CC–YJ, and TM–YJ. Orange, purple, and turquoise denote these three comparisons, respectively. (**b**) Distribution of nucleotide diversity (π) across genomic windows in Chicheng, Tumen, and Yanji. Red, green, and blue denote the three populations, respectively. (**c**) Observed ($H_{o}$) and expected ($H_{e}$) heterozygosity in the three populations. Blue and orange denote observed and expected heterozygosity, respectively. CC, Chicheng; TM, Tumen; YJ, Yanji.

Genome-wide $F_{\mathrm{ST}}$, nucleotide diversity (π), and heterozygosity were used to evaluate among-population differentiation and within-population diversity (Figure 2). Window-based $F_{\mathrm{ST}}$ distributions differed among the three population pairs (Figure 2a). The numbers of effective windows were 86,976 for CC–TM, 86,969 for CC–YJ, and 86,962 for TM–YJ. The mean window-level weighted Weir–Cockerham $F_{\mathrm{ST}}$ was highest between TM and YJ at 0.251 (95% CI: 0.247–0.255), followed by CC and TM at 0.179 (95% CI: 0.172–0.186), and was lowest between CC and YJ at 0.087 (95% CI: 0.082–0.092). The leave-one-chromosome-out estimates were stable across chromosomes, indicating that these differentiation patterns were not driven by any single chromosome.

The nucleotide-diversity analysis retained 86,965 effective windows in CC, 86,822 in TM, and 86,952 in YJ. Mean nucleotide diversity was comparable between CC and YJ, at $6.10\times 10^{-4}$ (95% CI: $5.70\times 10^{-4}$–$6.51\times 10^{-4}$) and $6.18\times 10^{-4}$ (95% CI: $5.93\times 10^{-4}$–$6.44\times 10^{-4}$), respectively (Figure 2b). TM showed substantially lower nucleotide diversity, with a mean π of $2.11\times 10^{-4}$ (95% CI: $1.88\times 10^{-4}$–$2.35\times 10^{-4}$).

Heterozygosity showed a pattern similar to nucleotide diversity (Figure 2c). YJ had the highest mean observed and expected heterozygosity ($H_{o}=0.289$ and $H_{e}=0.268$), CC had intermediate values ($H_{o}=0.218$ and $H_{e}=0.236$), and TM had the lowest values ($H_{o}=0.133$ and $H_{e}=0.131$). Observed heterozygosity was lower than expected heterozygosity in CC, approximately equal to it in TM, and higher than it in YJ.

### 3.4. Genomic Inbreeding and ROH Patterns

**Figure 3:**
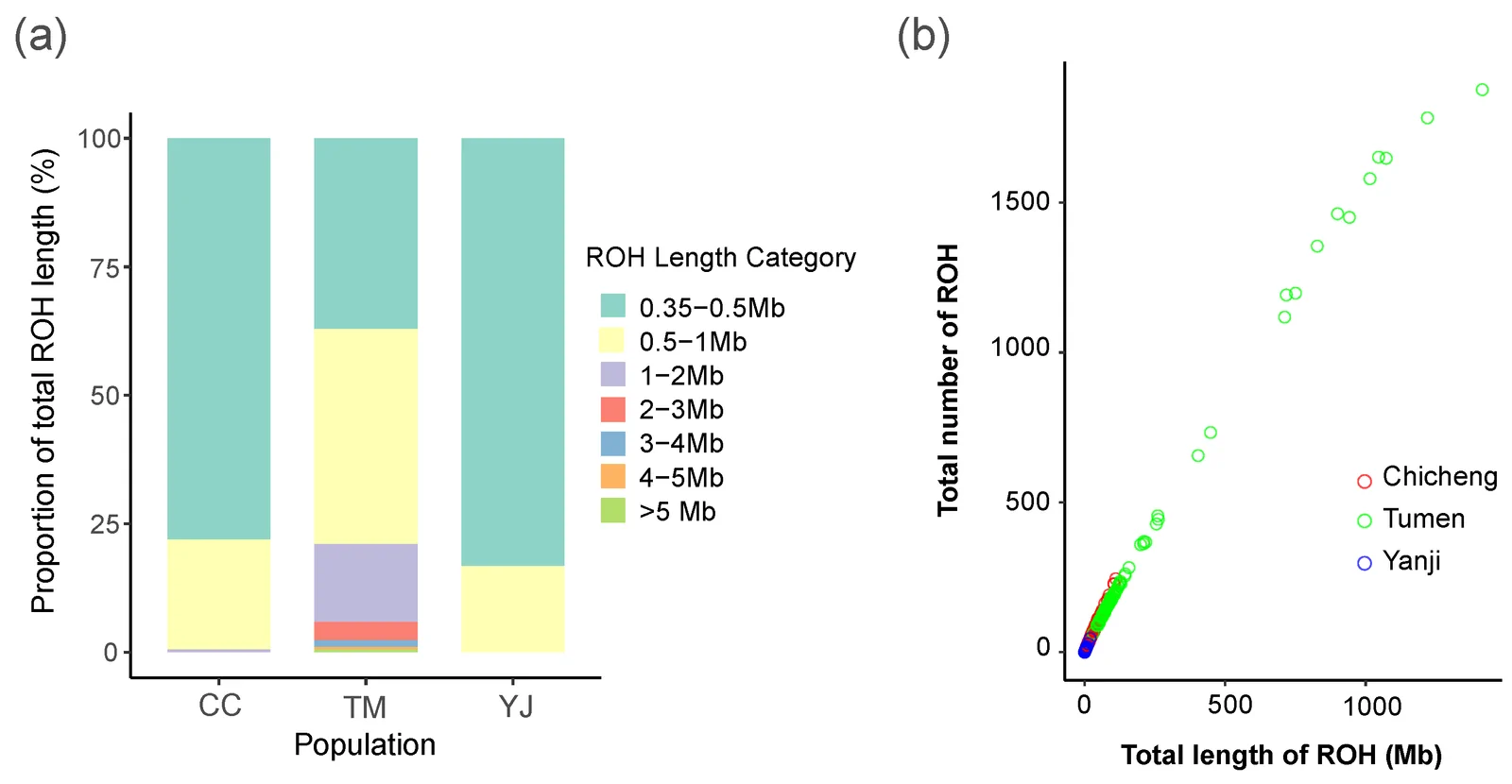
Runs of homozygosity (ROH) in three masu salmon populations. (**a**) Proportion of cumulative ROH length contributed by each ROH length class in Chicheng, Tumen, and Yanji. (**b**) Relationship between ROH number and cumulative ROH length for individual fish.

To evaluate genomic homozygosity and inbreeding, individual ROH number, cumulative ROH length, length-class composition, and the ROH-based genomic inbreeding coefficient ($F_{\mathrm{ROH}}$) were compared among populations (Figure 3; Table 1). Individual-level ROH traits differed markedly (Table 1; Figure S2), and the overall ROH burden followed the order TM > CC > YJ. TM had substantially higher ROH number, cumulative ROH length, and $F_{\mathrm{ROH}}$ than CC and YJ. In TM, means substantially exceeded medians and the ranges were wide, indicating that a small number of individuals with exceptionally high ROH burdens strongly influenced the population summaries. No ROH was detected in one CC individual or 44 YJ individuals, whereas every TM individual carried at least one ROH. Detailed summary statistics are provided in Table 1.

Nonparametric tests confirmed differences among the sampled populations in ROH number (H=326.05, p<0.001, $\epsilon^{2}=0.701$), cumulative ROH length (H=329.62, p<0.001, $\epsilon^{2}=0.709$), and $F_{\mathrm{ROH}}$ (H=329.62, p<0.001, $\epsilon^{2}=0.709$). All Holm-corrected pairwise comparisons were significant (p<0.001), with the ordering TM > CC > YJ and absolute rank-biserial correlations ranging from 0.919 to 0.998. The TM outlier-sensitivity analysis identified 17 individuals with $F_{\mathrm{ROH}}>0.0782$. After their exclusion, TM retained higher median ROH number (162), cumulative ROH length (86.13 Mb), and $F_{\mathrm{ROH}}$ (0.0319) than CC (29, 12.43 Mb, and 0.00460) and YJ (1, 0.35 Mb, and 0.00013). The omnibus and all pairwise comparisons remained significant (p<0.001), indicating that the observed ordering was not attributable solely to the highest TM values. Complete omnibus, pairwise, effect-size, and sensitivity-analysis results are provided in Supplementary Table S10.

ROH length composition also differed among populations (Figure 3a). CC and YJ were dominated by segments shorter than 1 Mb; the longest ROHs were 1.52 Mb in CC and 0.97 Mb in YJ. TM showed a broader length distribution containing short, intermediate, and long segments. ROHs longer than 5 Mb were detected only in TM, where the longest segment was 7.11 Mb.

The relationship between individual ROH number and cumulative ROH length showed a similar pattern (Figure 3b). CC and YJ individuals clustered mainly at low ROH numbers and short cumulative lengths, whereas TM showed a much broader distribution with several individuals exhibiting pronounced ROH accumulation.

### 3.5. Identification of Candidate ROH Islands

**Figure 4:**
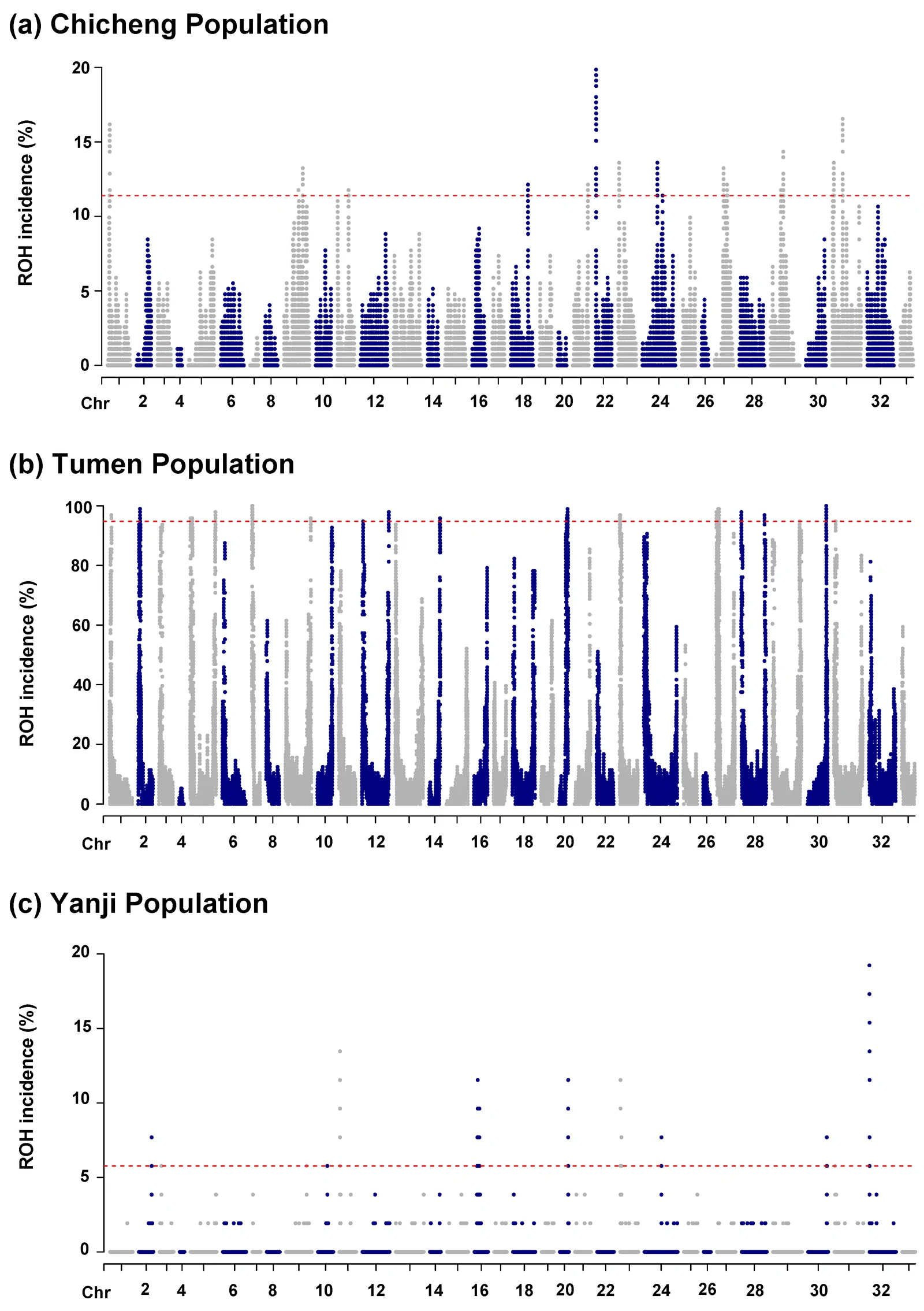
Genome-wide distribution of conditional ROH incidence and identification of candidate ROH islands in three masu salmon populations. (**a**) Chicheng. (**b**) Tumen. (**c**) Yanji. Blue and gray dots denote SNPs on alternating chromosomes. The red dashed line marks the population-specific top-0.1 % cutoff used for within-population prioritization.

To identify regions with high relative ROH incidence, we calculated the percentage of ROH-positive individuals in which each SNP was encompassed by an ROH. Incidence was therefore conditional on the presence of at least one detected ROH in an individual. For each population, SNPs in the top 0.1% of the incidence distribution, including all ties at the cutoff, were retained as high-incidence candidates. Adjacent candidate SNPs on the same chromosome and separated by ≤100 kb were merged into candidate ROH islands. The population-specific cutoffs shown in Figure 4 defined the within-population candidate sets. Figure 4a–c show the genome-wide conditional ROH-incidence profiles of CC, TM, and YJ, respectively.

A total of 67 candidate ROH islands were identified: 17 in CC, 31 in TM, and 19 in YJ. The number and genomic distribution of these islands differed markedly (Figure 4). TM had the largest number of candidate ROH islands and contained the broadest candidate regions, whereas CC and YJ had fewer and generally shorter islands. Of the 67 islands, 61 overlapped at least one reference gene (16 in CC, 27 in TM, and 18 in YJ). Detailed statistics for all candidate ROH islands are provided in Supplementary Table S6.

The fixed-prevalence sensitivity analysis confirmed that the absolute occurrence of ROH-enriched regions differed substantially among populations (Supplementary Table S8). At the 5% threshold, 129, 1130, and 19 candidate regions were retained in CC, TM, and YJ, respectively; at 10%, 25, 475, and 5 regions were retained. At 20%, 227 regions remained in TM, whereas none remained in CC or YJ. Maximum SNP-level prevalence reached 100% in TM but only 19.85% in CC and 19.23% in YJ. TM therefore retained more ROH-enriched regions than CC and YJ at each fixed threshold.

Table 2 lists the 58 candidate ROH islands containing at least one gene with a retained eggNOG-mapper gene symbol (16 in CC, 25 in TM, and 17 in YJ). The other three gene-overlapping islands lacked retained gene symbols and are reported in Supplementary Tables S6 and S7. Functional annotation identified 43, 51, and 73 non-redundant candidate gene symbols in CC, TM, and YJ, respectively. Their annotations encompassed growth and development, muscle organization, energy metabolism, ion transport, neural processes, cellular signaling, and immune regulation. Gene-level overlap was limited: CC and TM shared two genes, CC and YJ shared three, and TM and YJ shared four; no annotated candidate gene was common to all three populations. Thus, the gene content of candidate ROH islands was largely population-specific.

## 4. Discussion

### 4.1. Genetic Diversity and Population Differentiation

Genome-wide SNP data revealed clear genetic differentiation among the three masu salmon populations. PCA, ADMIXTURE, and NJ analyses consistently separated TM from the two landlocked cultured populations, CC and YJ, while also distinguishing CC from YJ (Figure 1). Nevertheless, CC and YJ were more closely related to each other than either was to TM. Pairwise $F_{\mathrm{ST}}$ estimates supported this pattern, with greater differentiation between TM and the cultured populations and lower differentiation between CC and YJ (Figure 2). The observed structure may reflect a combination of geographic origin, captive propagation history, and population management. Wild and cultured salmonid populations can diverge through ecological isolation, restricted gene flow, founder effects, broodstock composition, closed breeding programs, artificial selection, and translocation [23,43,44].

The populations also differed in LD decay and genetic diversity. TM exhibited faster LD decay but lower nucleotide diversity and heterozygosity than CC and YJ, indicating that LD- and diversity-based metrics capture different dimensions of population history. Previous reports indicate that hatchery facilities have been established in Jilin Province and that masu salmon are released annually for resource enhancement, including in the Tumen River system [45]. The reduced diversity observed in TM may reflect historical or recent demographic processes and could also be influenced by stock-enhancement practices, including a potential Ryman–Laikre effect [45,46,47,48]. Evaluating these possibilities will require information on hatchery broodstock size and genetic composition, variation in parental reproductive success, the survival and reproduction of released fish, and whether sampled individuals were naturally produced or hatchery-derived.

CC and YJ both retained higher nucleotide diversity and heterozygosity than TM. Farm records indicate that CC was established with fish obtained from the YJ source farm, consistent with the relatively low $F_{\mathrm{ST}}$ between these populations. Because CC was introduced only a few months before sampling, the observed differences in diversity and LD are unlikely to reflect long-term independent breeding or cumulative genetic erosion after establishment. Instead, they may reflect the number and reproductive contributions of founders, differences in family composition, or the sampling of different subsets from the source and recipient populations. Pedigree, parental-contribution, and relatedness data would help distinguish among these explanations.

### 4.2. ROH Patterns and Genomic Inbreeding

Compared with nucleotide diversity, heterozygosity, and LD decay, ROH patterns provide more direct information about genome-wide autozygosity. In particular, the accumulation of intermediate and long ROHs generally reflects more recent common ancestry or increased relatedness among individuals [17,18]. Differences in ROH number, cumulative ROH length, and $F_{\mathrm{ROH}}$ can therefore provide additional insight into the demographic and breeding processes underlying the contrasting diversity profiles of the three populations.

TM showed the greatest ROH accumulation, with substantially higher mean and median ROH number, cumulative ROH length, and $F_{\mathrm{ROH}}$ than CC and YJ. It was also the only population in which ROHs longer than 5 Mb were detected (Figure 3), indicating greater genome-wide homozygosity and autozygosity among the sampled TM fish. However, the large differences between means and medians show that a small number of individuals with exceptionally high ROH burdens substantially influenced the population averages. The coexistence of numerous short ROHs with intermediate and long segments is compatible with several overlapping processes, including genuine individual variation, reduced effective population size, unequal broodstock or family contributions, overrepresentation of related families, and mating among relatives. If the field-collected sample included hatchery-released fish or descendants of a limited number of broodstock, long-term stock enhancement may also have increased relatedness and ROH accumulation. Bottlenecks, limited broodstock, unequal family contributions, and kinship have all been associated with greater ROH accumulation in cultured or hatchery-supported salmonids [23,25]. Nevertheless, the extreme TM individuals do not demonstrate that recent inbreeding is population-wide, and the present data cannot resolve the relative contributions or timing of these processes.

ROH number, cumulative ROH length, and $F_{\mathrm{ROH}}$ were substantially lower in CC and YJ than in TM, indicating lower genomic inbreeding in the two cultured populations. CC was dominated by short ROHs, whereas YJ had the lowest overall burden. Given the short interval between the transfer of CC fish from the YJ source farm and sampling, differences between CC and YJ are unlikely to reflect long-term independent breeding or accumulated genetic change. They may instead reflect the representation and reproductive contribution of a subset of parental families or differences in family composition between the sampled groups. Because the sampled YJ fish were not necessarily the direct parents of the sampled CC fish, this explanation remains provisional without pedigree information. Overall, ROH patterns were broadly consistent with the population-structure and diversity results (Figure 1, Figure 2 and Figure 3): TM had the highest burden, CC had an intermediate burden dominated by short segments, and YJ had the lowest burden. Although ROH length distributions may contain information about both recent and historical common ancestry, the demographic processes responsible for these differences cannot be resolved from the present summaries alone.

### 4.3. Exploratory Functional Interpretation of Genes in Candidate ROH Islands

The limited gene-level overlap indicates that candidate ROH islands were largely population-specific. Only a few genes were shared between population pairs, and none was common to all three populations. Together with the marked differences in genetic structure and ROH patterns, this finding suggests that the genomic distribution of candidate islands reflects differences in genetic background, demographic history, and management among the populations.

Masu salmon has life-history-dependent maturation schedules: anadromous individuals generally mature after freshwater development, smoltification, and a marine growth phase, whereas some resident individuals, particularly males, can mature earlier as parr [1,49]. TM sampling intentionally prioritized large-bodied fish; however, age and migratory status were not recorded for the sampled individuals. Within this sampling context, TM candidate ROH islands contained genes annotated for ion regulation, neural signaling, sensory-system development, and energy metabolism. ATP1B2 is associated with the Na^+^/K^+^-ATPase complex and may contribute to ion regulation [50,51]. GRIN2A, GRIP1, and SDK1 participate in neural signaling or sensory-system development [52,53,54], whereas PDPR and ALAS1 are associated with mitochondrial metabolism and heme-dependent physiological processes [55,56]. These genes provide leads for investigating genomic variation associated with body size, developmental stage, and life-history differences. However, any relationships with migration and seawater adaptation remain hypothetical and require validation using age estimates, individual migration histories, and functional analyses.

CC and YJ showed little direct gene-level overlap but appreciable similarity in broad functional categories. Only ANAPC10, GAB1-like, and OTUD4 were shared (Table 2), with annotations related to cell-cycle regulation, intracellular signaling, and ubiquitin-mediated protein regulation, respectively. Nevertheless, both populations contained candidates associated with development, muscle or cytoskeletal organization, nutrient and energy metabolism, and neural regulation. CC-specific candidates included TCF7L2 and ZBTB16, associated with developmental regulation; SPEG, TRIM63, and MYO1C, associated with muscle organization; ATP5S and SLC43A2, associated with energy production or nutrient transport; and HTR3A, HTR3B, and FOXP2, associated with neural or behavioral processes. YJ-specific candidates included SOX11, NKX2-2a, PAX1, FOXA2, and HEY2, associated with developmental regulation; TTN, LAMA2, VGLL2, and LIMCH1, associated with muscle or cytoskeletal function; SLC16A10 and SLC25A36, associated with nutrient or mitochondrial transport; and CLSTN2, EPHA7, TAGAP, TRAF3IP2, and IL20RA, associated with neural or immune signaling. Representative genes such as TCF7L2, TTN, SPEG, ATP5S, and SLC16A10 have documented roles in development, muscle function, energy production, or nutrient transport [57,58,59,60,61,62,63], whereas HTR3A may contribute to serotonin-mediated behavioral and stress responses [64,65]. Thus, CC and YJ shared broad functional themes but differed in most specific candidate genes, potentially reflecting differences in founder composition, family representation, genetic drift, and culture history.

Differences in gene content provide biological context for the population-specific ROH patterns observed here. TM candidates were more frequently associated with ion regulation, neural processes, and energy metabolism, whereas candidates in CC and YJ more often involved development, muscle function, nutrient transport, and behavioral regulation. These patterns generate plausible hypotheses about biological differences among populations, but association, expression, and functional studies will be required to determine whether any candidate gene contributes directly to phenotypic variation.

### 4.4. Major Findings, Limitations, and Future Perspectives

Collectively, the analyses revealed pronounced differences in genetic structure, genetic diversity, and ROH distribution among the three masu salmon populations. TM was consistently separated from both cultured populations, while CC and YJ remained distinguishable despite their closer relationship and shared source history. The populations also differed in linkage disequilibrium and within-population diversity: CC had the highest LD, whereas TM had the fastest LD decay and the lowest nucleotide diversity and heterozygosity. ROH burden followed the order TM > CC > YJ. TM had the highest ROH number, cumulative ROH length, and $F_{\mathrm{ROH}}$, as well as the widest individual variation; CC had an intermediate burden dominated by short segments, and YJ had the lowest ROH-based genomic inbreeding. Candidate ROH islands and their gene contents showed little overlap among populations. TM candidates were more frequently associated with ion regulation, neural processes, and energy metabolism, whereas CC and YJ shared broad functional themes involving development, muscle organization, nutrient transport, and neural regulation but differed in most specific candidate genes.

These findings should be interpreted in light of limitations in the sampling design, variant dataset, and available background information. TM represents a single field-collected sample in which large-bodied fish were preferentially selected, but individuals could not be classified reliably by age, migratory status, or hatchery origin. The unequal sample sizes reflected the available quality-controlled collections rather than a balanced allocation, and no equal-size resampling analysis was performed. In addition, π, $H_{o}$, and $H_{e}$ were estimated from the retained polymorphic-SNP dataset rather than a callability mask containing invariant positions. CC and YJ comprised different sampled subsets rather than known parent–offspring groups, and systematic kinship or identity-by-descent analyses were not performed; consequently, the individual-level ROH tests were not adjusted for unknown kinship, and the broad TM distribution cannot by itself establish recent inbreeding as a population-wide condition. ROHs were detected using fixed PLINK thresholds, and the population-specific top-0.1% criterion provides within-population prioritization rather than a statistical test of selection. Candidate ROH islands may also arise through genetic drift, bottlenecks, founder composition, family overrepresentation, local recombination variation, or technical factors. Finally, candidate-gene interpretation depends on orthology-based annotation and published functions in other species; without matched phenotypic, expression, environmental, or experimental data, the functional and adaptive significance of these regions remains unresolved.

Future studies should include independent population replicates from multiple rivers, locations, and years, together with records of age, sex, body size, life-history type, stocking origin, and breeding history. Pedigree, kinship, identity-by-descent, and sensitivity analyses after kinship pruning would help distinguish population-wide patterns from family effects. Hidden Markov model-based ROH detection and demographic modeling could further separate recent inbreeding from historical population processes. Integrating these approaches with phenotypic, environmental, transcriptomic, and experimental data would enable more rigorous evaluation of candidate-gene functions.

## 5. Conclusions

Whole-genome comparisons revealed clear differences in genetic structure, diversity, linkage disequilibrium, and ROH distribution among the three masu salmon populations. TM was genetically distinct from the two cultured populations and showed the lowest nucleotide diversity and heterozygosity, and the highest ROH burden. CC and YJ were more closely related, but remained genetically distinguishable; both showed lower ROH burdens than TM, with YJ showing the lowest level. ROHs longer than 5 Mb occurred only in TM, indicating elevated homozygosity among the sampled fish.

Candidate ROH islands showed limited overlap among populations, and their annotated genes suggested different functional emphases: ion regulation, neural processes, and energy metabolism in TM, and development, muscle organization, nutrient transport, and neural regulation in CC and YJ. Representative candidate gene symbols included ATP1B2, GRIN2A, and PDPR in TM; TCF7L2, SPEG, and SLC43A2 in CC; and SOX11, TTN, and SLC16A10 in YJ. The present dataset provides a descriptive genomic baseline for the three sampled populations and a resource for future replicated monitoring and validation studies in masu salmon.

## Figures and Tables

**Table 1:** Individual-level summary statistics of runs of homozygosity (ROH) and ROH-based genomic inbreeding coefficients ($F_{\mathrm{ROH}}$) in three masu salmon populations.

Population	Trait	*n*	Mean ± SD	Median (Q1–Q3)	Range
Chicheng	ROH number	273	40.09 ± 37.91	29.00 (16.00–53.00)	0–245
(CC)	Cumulative ROH length (Mb)	273	17.373 ± 17.207	12.428 (6.462–22.607)	0–110.827
	$F_{\mathrm{ROH}}$ ($\times 10^{-3}$)	273	6.43 ± 6.37	4.60 (2.39–8.37)	0–41.05
	Mean ROH length (Mb)	272	0.424 ± 0.021	0.422 (0.410–0.437)	0.369–0.510
Tumen	ROH number	96	338.02 ± 435.47	174.00 (134.00–231.50)	11–1877
(TM)	Cumulative ROH length (Mb)	96	202.654 ± 292.524	91.535 (72.198–127.815)	4.791–1415.050
	$F_{\mathrm{ROH}}$ ($\times 10^{-3}$)	96	75.06 ± 108.34	33.90 (26.74–47.34)	1.77–524.09
	Mean ROH length (Mb)	96	0.546 ± 0.047	0.536 (0.521–0.553)	0.436–0.754
Yanji	ROH number	96	2.48 ± 6.12	1.00 (0.00–2.00)	0–46
(YJ)	Cumulative ROH length (Mb)	96	1.036 ± 2.654	0.354 (0–0.776)	0–19.627
	$F_{\mathrm{ROH}}$ ($\times 10^{-3}$)	96	0.38 ± 0.98	0.13 (0–0.29)	0–7.27
	Mean ROH length (Mb)	52	0.394 ± 0.031	0.396 (0.368–0.414)	0.351–0.461

Q1 and Q3 denote the first and third quartiles. For ROH number, cumulative ROH length, and $F_{\mathrm{ROH}}$, *n* includes all sampled individuals; individuals without detected ROHs were assigned zero. Mean ROH length was summarized only for ROH-positive individuals. CC contained one ROH-negative individual, YJ contained 44, and TM contained none.

**Table 2:** Candidate ROH islands containing at least one gene with a retained eggNOG-mapper gene symbol in three masu salmon populations.

Population	Chr.	Length (Mb)	Mean ROH Incidence (%)	Candidate SNPs	Annotated Gene Symbol(s)
Chicheng	1	0.404	15.78	189	SLC20A2
	9	0.178	11.76	312	YWHAE, MYO1C, SLC43A2, PITPNA, INPP5K, FBXO39, XAF1
	9	0.241	13.07	661	KIRREL3
	11	0.207	11.68	281	HTR3B, HTR3A, ZBTB16
	18	0.228	12.04	530	TCF7L2, VTI1A
	21	0.342	11.83	385	MAP4K5, PACS2, DCAF5, CDKL1, ATP5S, L2HGDH
	22	0.394	17.89	190	CAPRIN1, CSK, LMO2, SLC24A1
	23	0.322	13.16	37	ANAPC10, GAB1-like, OTUD4
	24	0.359	12.75	1112	BCL11A
	24	0.136	11.4	467	NFIA, FREM1
	27	0.309	12.42	654	TFEC, MDFIC, FOXP2
	27	0.19	11.95	183	EFNB3
	29	0.085	11.42	232	DCT, SOX21, ABCC4
	29	0.297	13.07	722	SPEG, TRIM63, COPS8, CTDSP1
	31	0.334	12.56	111	TMEM63B, CAPN11
	31	0.383	15.94	1065	VTI1A, TCF7L2
Tumen	1	0.365	96.37	135	SLC20A2
	2	0.592	97.24	244	GLG1, MRPS11, MRPL46, PDPR
	5	0.425	95.11	234	FBXL18, ACTB, FSCN1, PLK1, PDILT
	5	0.097	95.8	68	PLK1, FSCN1
	5	0.002	94.79	5	FBXL18
	5	0.393	95.79	218	TEKT5, TVP23A, GRIN2A
	5	0.165	95.55	59	SDK1
	5	0.374	96.79	140	ALAS1, SHISA5
	7	2.198	98.22	906	DIAPH3, TDRD3, PCDH20
	12	0.133	94.79	156	CSMD2
	12	0.37	97.26	128	HUNK, CADM2, SLC46A3, MIS18A, BACH1
	14	0.276	94.79	68	RBFOX1
	14	0.383	95.34	137	GRIN2A, ATF7IP2, NUBP1
	20	0.776	95.86	413	SLIT2, C4orf27, N4BP2, RHOH
	20	0.634	97.75	157	GSG2, LCORL, LIMCH1, FAM184B, TMEM192
	23	0.302	96.48	132	PLOD3, ATP1B2
	27	0.563	96.15	380	BRAF
	27	0.51	96.53	265	ARHGEF39
	27	0.483	94.79	165	KCNMB4, PTPRB, PTPRR
	27	0.484	96.52	196	GRIP1
	27	0.655	97.69	349	GRIP1, HELB
	28	0.379	96.89	278	PLOD2
	28	0.35	96.16	109	ST8SIA3
	29	0.109	94.79	110	CDKAL1
	31	0.359	94.79	126	CAPN11, CAPN2
Yanji	2	0.382	6.42	847	GYLTL1B
	3	0.349	5.77	98	PTPRF
	9	0.359	5.77	422	CLSTN2, SLC25A36, SPSB4
	10	0.401	5.77	774	TTN, LMLN
	11	0.404	9.92	112	LPAR1
	16	0.298	5.77	757	NKX2-2a, XRN2, NKX2-4, PAX1, FOXA2, CEBPZ, PRKD3
	16	0.38	6.78	903	SOX11
	16	0.452	9.09	885	ENPP1, SYTL3, EZR, TAGAP, PPP2R5A, DTL, INTS7, LPGAT1
	16	0.156	5.77	256	EPHA7
	16	0.36	5.77	621	FYN, TRAF3IP2, REV3L, MFSD4B, SLC16A10, RPF2, AMD1, CDK19, TAB2
	16	0.616	6.88	1733	SLC35D3, HDDC2, ARHGAP18, LAMA2, L3MBTL3, TMEM200A, EPB41L2, AKAP7, IL20RA, PEX7, HEY2, TPD52L1, RNF217, NKAIN2
	16	0.561	8.11	1250	VGLL2, RFX6, UNC93A, TDRD15, TTLL2, SLC35F1, NUS1, GOPC, DCBLD1, MLLT4, PLG
	20	0.479	8.46	68	GSG2, LCORL, LIMCH1, FAM184B
	23	0.415	6.96	60	ANAPC10, GAB1-like, OTUD4
	24	0.277	6.51	488	EML4, TRIM35
	31	0.364	5.77	50	PRR18
	32	0.44	17.3	155	VPS39, NUMB, GALNT16

## Data Availability

The quality-controlled VCF dataset containing 6,220,980 SNPs from 465 individuals is available from Zenodo at DOI: https://doi.org/10.5281/zenodo.21736114.

## Supplementary Materials

The following supporting information can be downloaded at https://www.mdpi.com/article/10.3390/biom16081187/s1, Figure S1: ADMIXTURE results for K = 2–5; Figure S2: Individual-level distributions of ROH number, cumulative ROH length, and $F_{\mathrm{ROH}}$; Table S1: Sampling information; Table S2: Population-level clean-read quality-control summary; Table S3: Sample-level clean-read quality-control metrics; Table S4: Chromosome-specific SNP distribution and density; Table S5: SNP genomic-category summary; Table S6: Candidate ROH island statistics; Table S7: Genomic context and eggNOG-mapper annotations of candidate genes; Table S8: Fixed-prevalence sensitivity analysis of candidate ROH-enriched regions; Table S9: Numerical summary of LD decay; Table S10: Nonparametric comparisons and TM outlier-sensitivity analysis of individual ROH traits.
