# Supplementary material for: Genetic Diversity and Runs of Homozygosity in Three Masu Salmon (Oncorhynchus masou) Populations Based on Whole-Genome Resequencing Data
Source: Biomolecules. 2026 Aug 14;16(8):1187. doi: 10.3390/biom16081187 (PMC13510946; doi:10.3390/biom16081187)
Supplement: Supplementary file 1 [file biomolecules-16-01187-s001.zip › Figure_S1_ADMIXTURE_K2-K5.pdf]

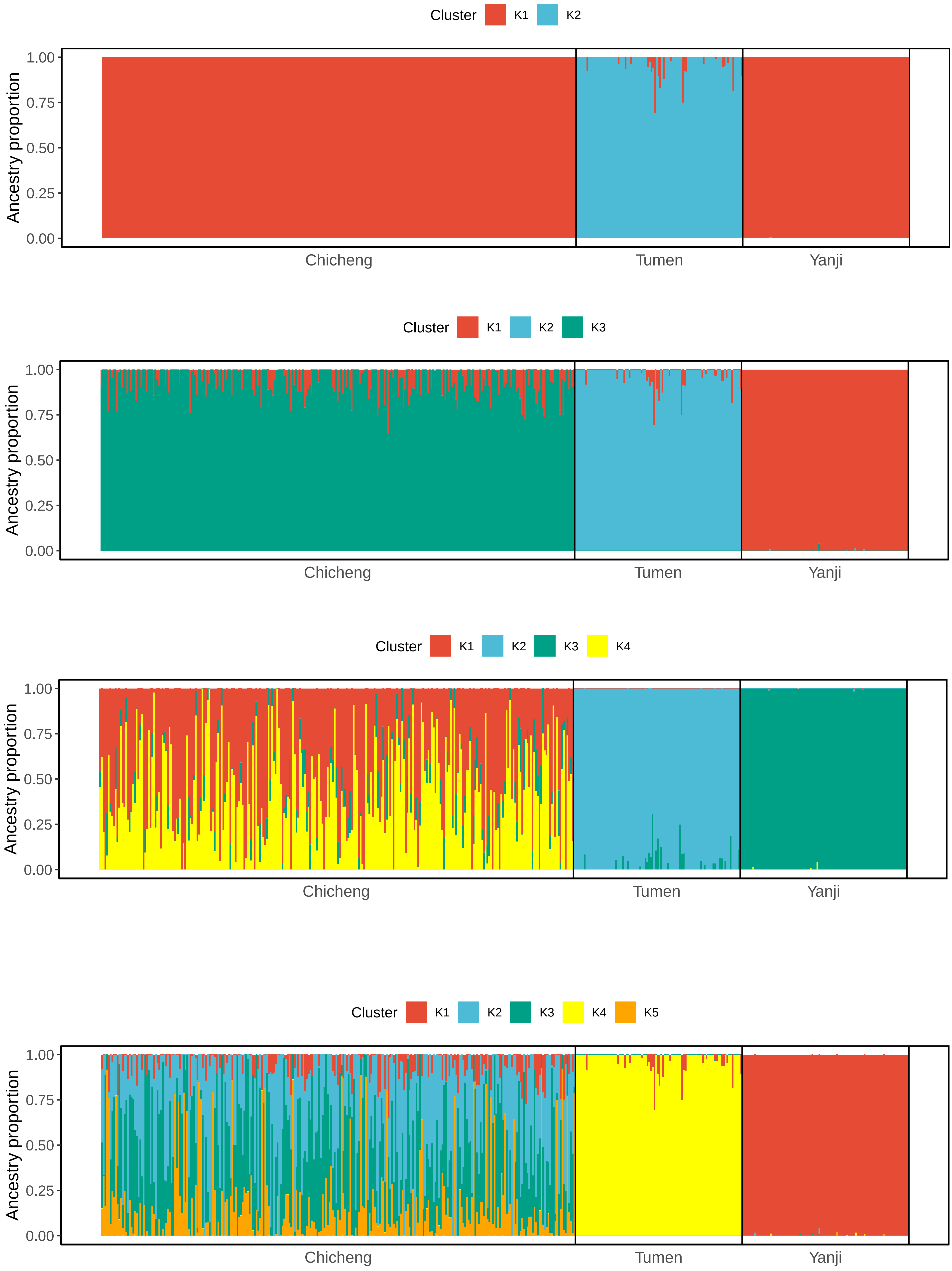
Cluster K1 K2
Ancestry proportion
1.00
0.75
0.50
0.25
0.00
Chicheng
Tumen
Yanji
Cluster K1 K2 K3
Ancestry proportion
1.00
0.75
0.50
0.25
0.00
Chicheng
Tumen
Yanji
Cluster K1 K2 K3 K4
Ancestry proportion
1.00
0.75
0.50
0.25
0.00
Chicheng
Tumen
Yanji
Cluster K1 K2 K3 K4 K5
Ancestry proportion
1.00
0.75
0.50
0.25
0.00
Chicheng
Tumen
Yanji
