# Supplementary material for: Genetic Diversity and Runs of Homozygosity in Three Masu Salmon (Oncorhynchus masou) Populations Based on Whole-Genome Resequencing Data
Source: Biomolecules. 2026 Aug 14;16(8):1187. doi: 10.3390/biom16081187 (PMC13510946; doi:10.3390/biom16081187)
Supplement: Supplementary file 1 [file biomolecules-16-01187-s001.zip › Figure_S2_Individual_ROH_Distributions.pdf]

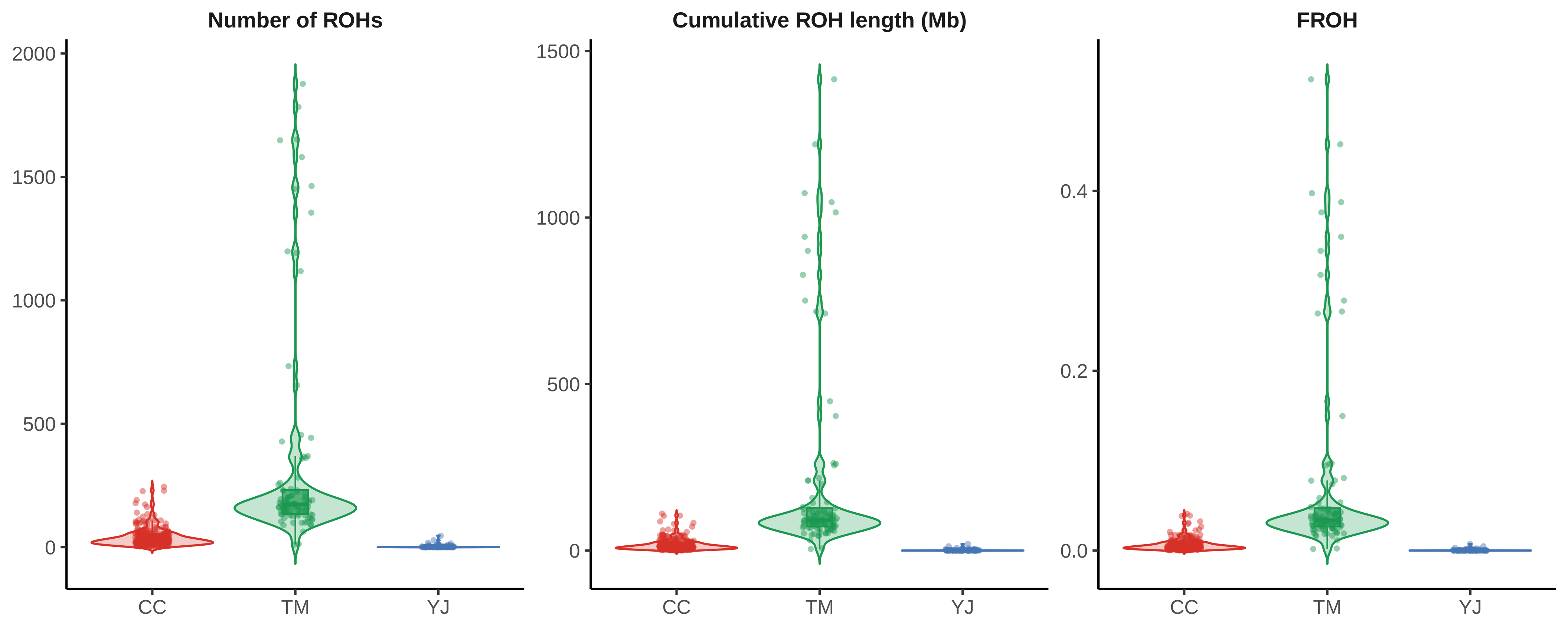
Number of ROHs
Cumulative ROH length (Mb)
FROH
2000
1500
1000
500
0
0.4
0.2
0.0
CC
TM
YJ
